# Microlesion Effect Induced by Electrode Implantation in the Posteroventral Globus Pallidus Interna for Severe Dystonic Tics

**DOI:** 10.5334/tohm.837

**Published:** 2024-01-18

**Authors:** Galih Indra Permana, Takashi Morishita, Hideaki Tanaka, Hitoshi Iida, Shinsuke Fujioka, Hiroshi Abe

**Affiliations:** 1 Department of Neurosurgery, Fukuoka University Faculty of Medicine, Fukuoka, Japan; 2 Department of Neurosurgery, Dr. Moewardi General Academic Hospital, Central Java, Indonesia; 3 Department of Psychiatry, Fukuoka University Faculty of Medicine, Fukuoka, Japan; 4 Department of Neurology, Fukuoka University Faculty of Medicine, Fukuoka, Japan

**Keywords:** Severe dystonic tics, deep brain stimulation, microlesion effect, globus pallidus interna

## Abstract

**Background:**

Tourette syndrome (TS) is a neurologic condition characterized by motor and phonic tics. Dystonic tics, including blepharospasm, are considered atypical or unusual in severe TS.

**Case Report:**

We report a severe case of TS with facial dystonic tics resembling blepharospasm in which the microlesion effect and a sustained therapeutic effect was observed with bilateral globus pallidus interna (GPi) deep brain stimulation (DBS).

**Discussion:**

Bilateral GPi DBS can be beneficial for blepharospasm-like tics and severe symptoms of TS. The improvements seen can be explained by the microlesion effect induced by DBS lead placement in the GPi.

## Introduction

Tourette syndrome (TS) is a neurologic condition characterized by motor and phonic tics. It may accompany obsessive compulsive disorder (OCD), attention deficit hyperactivity disorder (ADHD), poor impulse control, and other behavioral issues. The motor and phonic tics tend to be relatively brief and intermittent [1, 2], although dystonic motor tics are more prolonged. Clonic tics typically start abruptly and quickly, but slower tics may cause a briefly prolonged abnormal posture (dystonic tics) or an isometric contraction (tonic or isometric tics) [2, 3]. Dystonic tics, including blepharospasm, are considered atypical or unusual in severe TS. A potential treatment for malignant or medically intractable TS is deep brain stimulation (DBS), but no consensus has been reached about the target anatomical locations for DBS [4]. The globus pallidus interna (GPi) has been increasingly used as a target in patients with disabling tics because this site has long been targeted in the treatment of other hyperkinetic movement disorders. We report a severe case of TS with facial dystonic tics resembling blepharospasm in which the microlesion effect and a sustained therapeutic effect was observed with bilateral posteroventral GPi (pvlGPI) DBS.

## Case Report

A 26-year-old man with severe TS complained of difficulty opening his eyes. He was diagnosed with TS at 10 years of age and had tried multiple medications including trihexyphenidyl, clonazepam, risperidone, olanzapine, and aripiprazole. However, his symptoms were refractory to medication, so he was referred to our department to consider surgical treatment. His past medical history was not significant for other neuropsychiatric disorders. On examination, he had frequent blepharospasm-like tics accompanying premonitory urge. Other signs and symptoms of his motor and phonic tics were mild, such as twitching of the mouth and trunk and cough. His total Yale Global Tic Severity Scale (YGTSS) severity score was 20 and the impairment score was 50. His daily living activities were severely impaired by the blepharospasm-like tics, and thus DBS therapy was indicated. GPi DBS for both tics and blepharospasm (Meige syndrome) has been reported (1), but there are very few reports on targeting the anteromedial part of the GPi or centromedian thalamic nucleus for blepharospasm. Since we could not completely rule out the possibility that his abnormal blinkings are due to dystonia complicated with TS, we selected the pvlGPi as a DBS target in this patient. He was only on trihexyphenidyl at the timing of surgery.

**Figure 1:**
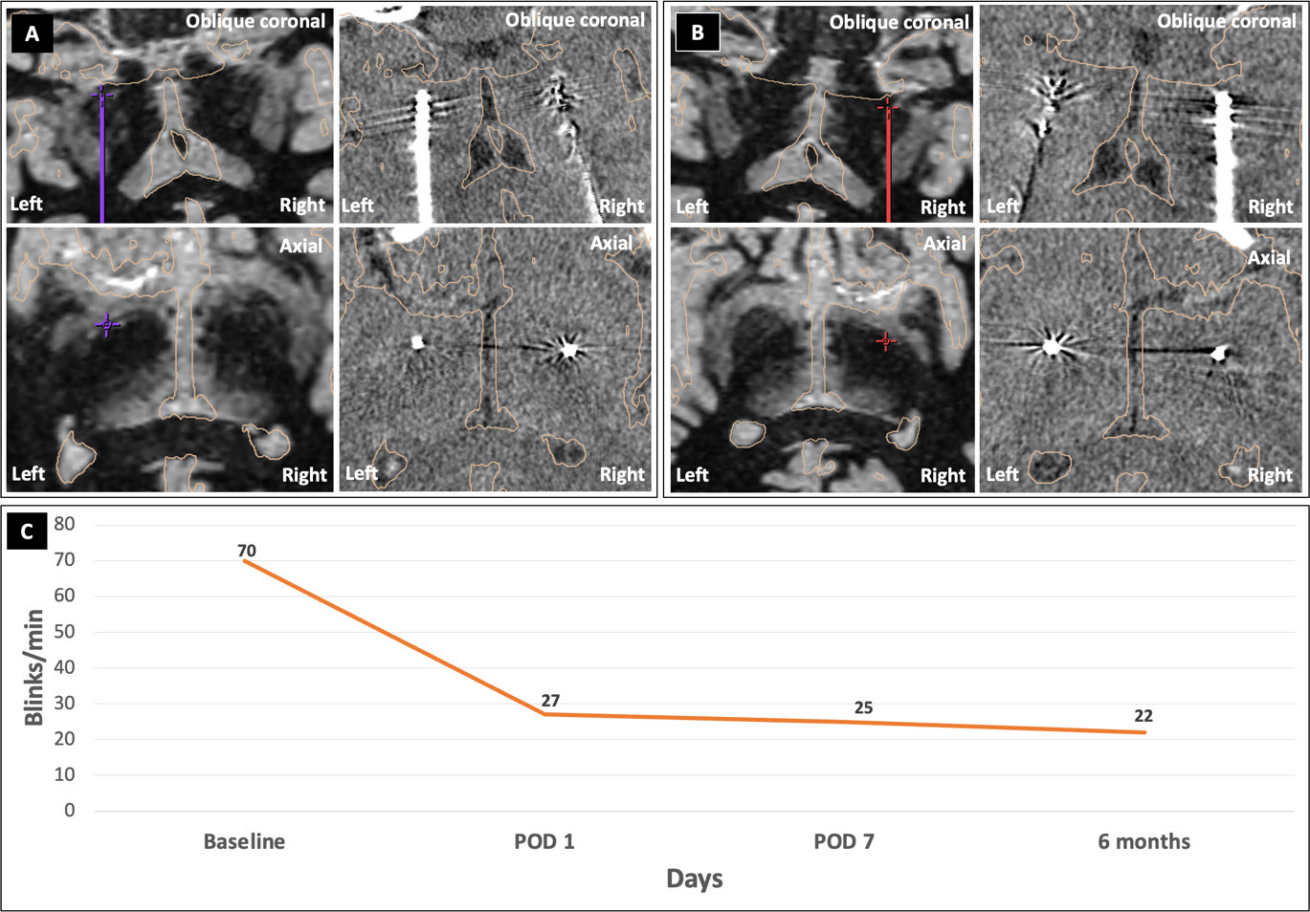
**Pre- and postoperative imaging and clinical course**. Fast gray matter acquisition T1 inversion recovery magnetic resonance imaging sequences (oblique coronal and axial) to plan lead positioning preoperatively and computed tomography scans (coronal and axial) to evaluate lead placement postoperatively in the **(A)** left posteroventral globus pallidus interna (pvlGPI) and **(B)** right pvlGPI. **(C)** Change in blepharospasm-like tic frequency from before surgery to 6-months after bilateral pvlGP deep brain stimulation.

High-resolution MRI sequences were fused using software for stereotactic planning to determine placement of the DBS lead. The mid-commissural point was used to center the planes and use Cartesian coordinates in the surgery. The route was decided considering the pvlGPi structures that could be seen and a safe insertion angle. The target coordinates of the left pvlGPi were X = 20.5 mm to the left, Y = 4.0 mm anterior, Z = 7.0 mm inferior, AC-PC angle = 60.3°, and center line angle = 10.1° to the left; those of the right pvlGPi were X = 21.0 mm to the right, Y = 4.0 mm anterior, Z = 5.5 mm inferior, AC-PC angle = 60.5°, and center line angle = 11.1° to the right (Table 1, Figure 1). We implanted the pvlGPi DBS lead (model B33015; Medtronic, Minnesota, MN) under local anesthesia and then the pulse generator (model B35200; Medtronic) under general anaesthesia on the same day. Lead placement was confirmed on postoperative computed tomography (Figure 1).

**Table 1:** DBS coordinates and programming parameters.


DBS COORDINATES	X	Y	Z	AC-PC ANGLE	CENTER LINE ANGLE

Left pvlGPi	20.5 mm left	4.0 mm anterior	7.0 mm inferior	60.3°	10.1°

Right pvlGPi	21.0 mm right	4.0 mm anterior	5.5 mm inferior	60.5°	11.1°


The blink frequency was determined (times/min) from video recordings of the patient made at baseline, on postoperative day (POD) 1 and POD 7, and at 6 months after the surgery (Figure 1). The blink frequency was improved from 70 times/min at baseline to 27 times/min on POD 1. DBS was initiated on POD 7 after evaluating the blink frequency (25 times/min). The patient was discharged on POD 9, and followed up at clinic once a month.

We initially activated the second ventral contacts (contact 1 on the left; contact 9 on the right) at 60 microsec, 130 Hz, and 1.5 mA, and the stimulation intensity was gradually increased as the microlesion effects waned. The DBS parameters at the 6-month follow-up were as follows: left, 1– (0.7 mA), 2– (1.6 mA), 3– (0.5 mA), Case+, 60 microsec, 135 Hz; right, 9a–v(0.5 mA), 9c–v(0.3 mA), 10a–v(0.7 mA), 10b–v(0.3 mA), 10c–(0.3 mA), 11–v(0.5 mA) Case+, 60 microsec, 135 Hz (Table 2). At the 6-month follow-up, blink frequency was slightly improved to 22 times/min, with the YGTSS severity and impairment scores substantially improved to 4 and 0, respectively. He stopped taking trihexyphenidyl after evaluation on POD 7, and was able to return to work without uncomfortable feelings in his blinking behavior after discharge. There were no adverse effects and the patient was satisfied with the therapeutic effects.

**Table 2:** **The stimulation parameters at each visit**. Stimulation areas and amplitudes were gradually increased.


TIME POINT	LEFT				RIGHT			
	
	CATHODE (AMPLITUDE (mA))	ANODE	PW (µsec)	FREQUENCY (Hz)	CATHODE (AMPLITUDE (mA))	ANODE	PW (µsec)	FREQUENCY (Hz)

**1 mo**	2 (1.5)	Case	60	130	10 (1.5)	Case	60	130

**2 mo**	2 (1.8)	Case	60	130	10 (1.8)	Case	60	130

**3 mo**	1 (0.3), 2 (1.5)	Case	60	130	9 (0.3), 10 (1.5)	Case	60	130

**4 mo**	1 (0.7), 2 (1.6)	Case	60	135	9a (0.5), 9c (0.3), 10a (0.7), 10b (0.3), 10c (0.3)	Case	60	135

**5 mo**	1 (0.7), 2 (1.6), 3 (0.5)	Case	60	135	9a (0.5), 9c (0.3), 10a (0.7), 10b (0.3), 10c (0.3)	Case	60	135

**6 mo**	1 (0.7), 2 (1.6), 3 (0.5)	Case	60	135	9a (0.5), 9c (0.3), 10a (0.7), 10b (0.3), 10c (0.3), 11 (0.5)	Case	60	135


## Discussion

Motor tics typically consist of sudden, abrupt, transient, repetitive, and coordinated (stereotypical) movement that may resemble gestures and mimic fragments of normal behavior, vary in intensity, and are repeated at irregular intervals [1, 5, 6]. The present case showed a severe blepharospasm-like dystonic tics affecting the quality of life and activities of daily living. The patient’s specific needs must be considered when developing strategies for treatment. DBS is, therefore, a potential treatment reserved for patients with severe TS that has been refractory to medical treatment [3, 7,8,9,10]. Although the pathophysiology of tics is not fully understood, it is thought that tics are due to dysfunction in the basal ganglia–thalamo–cortical loops, which can be favourably modified by DBS [11, 12].

Concerning the DBS target, no significant differences between targets have been detected according to the International Deep Brain Stimulation Database and Registry [4], and the recent European guidelines recommended no specific target [4]. The most common target in TS with dystonic tics is the thalamic centromedian-parafascicular (CM-pf) complex [6]. Stimulation of the anteromedial GPi has resulted in slightly but non-significantly higher improvement rates compared with thalamic DBS followed by the pvlGPi [10, 11]. In our case, the blepharospasm and other severe symptoms of TS have improved after bilateral pvlGPi DBS. The evaluation that was performed 6 months after DBS showed improvement of the YGTSS scores. We consider that patient could achieve almost normal blink rate in the 6 months following surgery as the mean spontaneous blink frequencies in TS patients and healthy subjects have been reported to be 13.1–26.5 times/min and 33.6–44.7 times/min [13].

We selected bilateral pvlGPi DBS in the present case, which markedly improved the patient’s dystonic tics immediately after the surgery without even initiating stimulation. This suggests that bilateral pvlGPi DBS can be beneficial for severe symptoms of TS as well as blepharospasm. The improvements seen can be explained by the microlesion effect induced by DBS lead placement in the GPi. This effect is considered to result from edema, microhemorrhage, and the disruption of cells and/or fibers along the trajectory of DBS electrodes [5, 14, 15]. Our case indicates that the microlesion effect could potentially indicate optimal lead placement given that the microlesion effect was evident immediately after the surgery, and sweet spots for tic suppression may exist in the GPi.

This case study showed that, in cases of severe TS, the microlesion effect may appear as an immediate improvement following the implantation of a DBS lead in the GPi. Based on our findings, patients with severe TS can have sweet spots for tic suppression, and the identification of sweet spot for microlesion effect in the GPi may guide the DBS programming. Additional research involving more patients is necessary.
